# Characterization of Electron Beam-Induced Polymerization of Isodecyl Methacrylate, Benzyl Methacrylate, and Their Equimolar Mixture Based on Monomer Properties

**DOI:** 10.3390/polym18030368

**Published:** 2026-01-29

**Authors:** Ilia Antonov, Mikhail Mikhailenko, Tatyana Shakhtshneider, Artem Ulihin, Maxim Zelikman, Alexandr Bryazgin, Ilia Eltsov

**Affiliations:** 1 Institute of Solid State Chemistry and Mechanochemistry SB RAS, Novosibirsk 630090, Russia; mikhailenko@solid.nsc.ru (M.M.); shah@solid.nsc.ru (T.S.); a.ulihin@gmail.com (A.U.); zelikman@solid.nsc.ru (M.Z.); 2 Budker Institute of Nuclear Physics SB RAS, Novosibirsk 630090, Russia; a.a.bryazgin@inp.nsk.su; 3 Laboratory for Synthesis and Physicochemical Studies of New Composite Catalysts, General Chemistry Section Novosibirsk, Novosibirsk State University, Novosibirsk 630090, Russia; eiv@fen.nsu.ru

**Keywords:** benzyl methacrylate, isodecyl methacrylate, electron beam, polymerization, copolymer synthesis

## Abstract

The study examined the polymerization of methacrylic acid derivatives with bulky substituents (isodecyl-, benzyl-), as well as the synthesis of their copolymer through radiation initiation followed by thermal treatment. It has been discovered that the polymerization rate in equimolar monomer mixtures substantially surpasses the rate in pure monomers. We hypothesize that the substantial degree of association with the liquid monomer hinders structural rearrangements preceding polymerization. We tested this hypothesis by employing various analytical methods.

## 1. Introduction

Developing smart materials is a promising avenue for methacrylate polymer application. Smart materials are those that can alter their characteristics in response to environmental conditions such as temperature, light, pH, and other external factors. For instance, at a specific pH level, materials capable of self-healing [1] can repair both micro- and macro-damages [2,3]. This phenomenon is because the molecules of the substance can move and eventually mend broken bonds. These mobile chains consist of chain segments where one end is not linked to the network. To develop self-healing materials, polymer hydrogels are frequently utilized.

Another appealing application for smart materials is the design of systems with the regulated release of physiologically active substances into solution [4]. The heterocyclic methacrylate polymer system has been investigated as a biomaterial to promote bone and cartilage regeneration, as well as a drug delivery system. This was accomplished by polymerizing a blend of polyethyl methacrylate and tetrahydrofurfuryl methacrylate monomer at room temperature [5].

Polymers based on methacrylic acid esters with bulky substituents are useful not only as drug delivery systems [5] and materials for other medical applications [6], but they are also applicable as adhesives [7], thermoplastics [8], self-healing materials [9], materials for electron-optical devices [10], sensors, and surfactants [11]. Additionally, they can be employed in non-printing surface lithography to create computer memory components with random-access memory [12] and composites with a high heat capacity and thermal conductivity [13].

The same techniques that have been previously studied [14,15] for the polymerization of methyl methacrylate can be applied to the polymerization of methacrylic acid esters with bulky substituents. Yet, specific features during polymerization depend on the particular structure of monomers with bulky substituents.

To conduct bulk polymerization, a monomer-soluble radical initiator is typically added to the pure monomer in its liquid state, and heated or exposed to radiation. Free-radical bulk polymerization of vinyl monomers (such as vinyl acetate, styrene, ethylene, and derivatives of acrylic and methacrylic acids) is known to exhibit the phenomenon of autoacceleration [16]. Up to a certain monomer conversion value, the free-radical polymerization of these monomers can be explained by the classical theory. The polymerization process autoaccelerates once the monomer conversion reaches a critical degree [17]. Overheating of the reaction mixture, a decrease in the rate of chain termination, an increase in viscosity, and a decrease in thermal conductivity are the causes of this phenomenon [18]. The kind of monomer, the kind and concentration of the initiator, the temperature, and additional reaction parameters all affect the start of autoacceleration and its intensity. This phenomenon is most apparent in the bulk polymerization of methyl methacrylate [19].

A disadvantage of conventional polymerization methods is that the end products often contain residual agents, which often impair their optical and electronic properties [20,21]. Removal of the initiator and its decomposition products from polymers is a complex and expensive procedure [22,23]. The application of physical initiation has made it possible to obtain high-molecular-weight polymers in bulk and in solution. These polymers enable the synthesis of composites with more fillers compared to composites produced using traditional methods [24,25].

One of the methods of physical initiation is radiation initiation. Initiation is carried out by irradiation [26] or an internal radiation source [27]. In addition to polymerization, radiation initiation can be used as a way to modify materials [28]. Depending on the absorbed radiation doses, two distinct processes occur: the creation of new inter-chain and intra-chain chemical bonds (i.e., polymerization and/or crosslinking) and the breaking of the chemically bonded structure, or degradation, which is strongly dependent on the environmental atmosphere, i.e., oxidative degradation, and on the dose rate [29].

Previously, we conducted a screening study on the synthesis of individual polymers and copolymers of different compositions using the method of radiation initiation and subsequent thermal treatment. The monomers used were methacrylic acid esters: ethylhexyl, cyclohexyl, benzyl, isodecyl, and isobornyl methacrylate. We needed a copolymer with high adhesion to metal, and the composite matrix had to accommodate a large amount of filler. The isodecyl methacrylate-benzyl methacrylate system was selected for more detailed studies [30]. Based on this copolymer, we managed to obtain a coating with over 30 vol. % tungsten where no percolation conductivity was present [31].

The aim of this work was to investigate the characteristics of radiation-induced polymerization of methacrylic acid esters with big substituents (isodecyl, benzyl) and the synthesis of a homogeneous copolymer of isodecyl methacrylate and benzyl methacrylate.

## 2. Materials and Methods

Ethyl acetate (EKOS-1 Staraya Kupavna, Russia), ethanol, tetrahydrofuran (THF) (Reachim JSC, Moscow, Russia), isodecyl methacrylate (Evonik Darmstadt, Germany), benzyl methacrylate (Evonik Darmstadt, Germany), and deuterated chloroform 99.8% D (Solvex-D Moscow, Russia) were used in the work.

THF was purified by distillation under reduced pressure using a rotary evaporator. Methacrylates were purified as follows. A total of 100 mL of 5% sodium hydroxide solution were added to 100 mL of methacrylate, stirred, and after 1 h the aqueous solution was separated off using a separating funnel. Following this, 100 mL of distilled water were then added to 100 mL of the organic portion, stirred, and after 1 h the aqueous solution was separated off. The procedures were repeated until the pH of the aqueous portion was neutral. The organic portion was then dried from residual water in a vacuum oven at low pressure at a temperature of 50 °C.

### 2.1. Synthesis of Individual Polymers

Polymerization was carried out using the radiation initiation method, in which benzyl methacrylate and isodecyl methacrylate were treated with a beam of accelerated electrons using the ILU-6 accelerator (INP SB RAS, Novosibirsk, Russia) with an electron energy of 2.4 MeV. The processing conditions were as follows: beam pulse current 320 mA, pulse duration 0.6 ms, pulse repetition rate 2.5 Hz, and sample movement speed under the accelerator outlet window 2 cm/s. The irradiation dose for one pass under the accelerator exit window was 2.5 kGy. The total treatment dose was between 10 and 160 kGy (J/g). The polymerization process of initiated monomers could occur at a temperature higher than 60 °C. Subsequently, the polymerization process was carried out at a temperature of 70, 80, 90, 100, and 110 °C for 0.5–144 h. The polymer, following the heat treatment, was purified by dissolving it in ethyl acetate and precipitating it with ethyl alcohol, while residual monomer remains dissolved. The process was repeated three times. The product was then dried for 10 h at a temperature of 50 °C and reduced pressure.

### 2.2. Synthesis of Copolymer

The conditions for obtaining a copolymer with the uniform monomer distribution were studied using the reaction mixtures of monomers with a molar ratio of benzyl methacrylate to isodecyl methacrylate of 1:1 (1:1.285 by weight). The monomers were individually subjected to electron beam treatment before the experiments. This was due to the complexity of the two-monomer system. Therefore, only by individually treating each monomer can an equimolar mixture of initiated monomers be achieved. Otherwise, the ratio of initiated monomers in the mixture will vary widely. The mixture of irradiated monomers was placed in a thermostat under conditions similar to those for individual monomers. At the initial stage of polymerization, samples were taken from which a low molecular weight copolymer was isolated. This copolymer was examined using NMR spectroscopy (^1^H, ^13^C) to determine the ratio of isodecyl and benzyl units. The conversion degree of polymer samples for analysis was less than 5%.

After the heat treatment, the copolymer was purified using the same procedure as for individual polymers. The yield of copolymer was at least 80%.

### 2.3. Characterization

The polymerization kinetics were studied by gel permeation chromatography (GPC) on a chromatography system with a refractometric detector (Knauer, Berlin, Germany) and a PLgel 20 μm MIXED-A column, 300 × 7.5 mm (Agilent, Santa Clara, CA, USA), calibrated with a set of polystyrene calibration standards (Mp~900–1,500,000) (Agilent). Solvent: THF; flow rate: 1 mL/min.

Nuclear magnetic resonance studies were performed using an AVANCE III 500 spectrometer (Bruker, Bremen, Germany) at a frequency of 500 MHz (^1^H). The spectra of individual monomers in situ and DMSO were obtained at temperatures of 295–385 K in steps of 10 K. The spectra of the copolymer were recorded in CDCl_3_ at 295 K.

The electrochemical properties were measured using a special cell containing two platinum wire electrodes placed in the sample. The impedance spectra of the samples were recorded in the stepwise temperature mode. The heating rate was 20 degrees/hour. The measurements were carried out with a Zive SP2 electrochemical station (WonATech Co., Ltd., Seoul, Republic of Korea) in the frequency range of 0.5–1 MHz’s

The dynamic viscosity of solutions in ethyl acetate was investigated at a temperature from 25 to 65 °C using a rotational viscometer LVDV-II+ (Brookfield, New York, NY, USA).

The dielectric loss tangent was calculated from the values of the real and imaginary parts of the conductivity using the formula tg (d) = −Z”/Z’. Conductivity measurements were executed in a two-electrode cell with platinum electrodes in a vacuum of 5 × 10^−2^ Torr within the temperature range of 25–100 °C with alternating current using an MNIPI E7-25 immittance meter in the frequency range of 30–1 MHz. Conductivity values were calculated by analyzing impedance hodographs in Nyquist coordinates Z”-Z’.

The data on the radial distribution of monomers were derived from classical molecular dynamics computer simulations using the GROMACS 2025.4 software package. The molecular models were derived from the Protein Data Bank database. The Lennard–Jones and Coulomb interaction energies were calculated using GROMACS 2025.4 utilities.

## 3. Results and Discussion

### 3.1. Analysis of the Reactivity of Monomers and Their Mixtures

**Figure 1:**
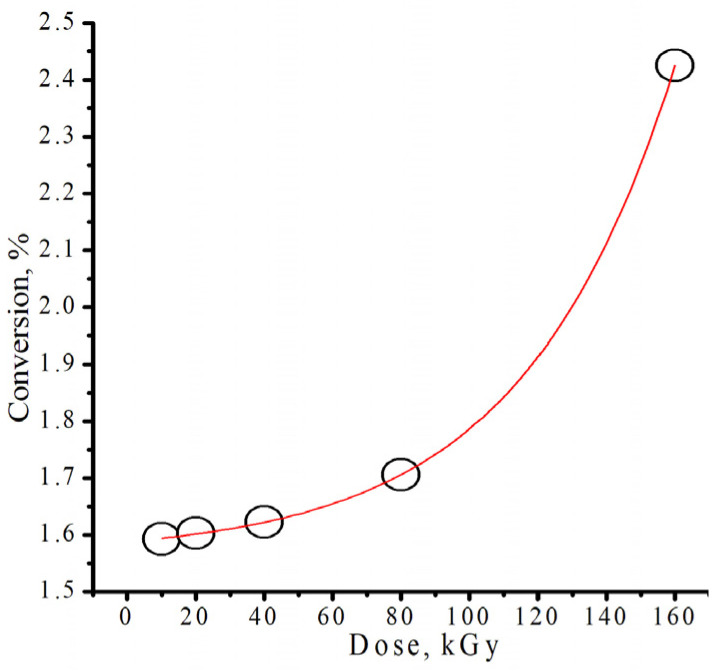
The relationship between conversion of poly(benzyl methacrylate) and the irradiation dose.

To investigate the effect of different doses of radiation on the yield of the polymerization product, benzyl methacrylate (2 mL) was irradiated with doses ranging from 10 to 160 kGy. Figure 1 illustrates how the radiation dose affects the conversion of polybenzyl methacrylate following 2 h of heat treatment at 70 °C. It is exponentially dependent. Analysis of the impact of radiation doses on the isodecyl methacrylate polymerization process yielded similar findings, though polymerization did occur for doses below 20 kGy. A dose of 20 kGy was selected for subsequent studies. This choice was justified by the fact that with a high dose of radiation there is a risk of overheating the reaction mixtures and destroying the polymer [32].

**Figure 2:**
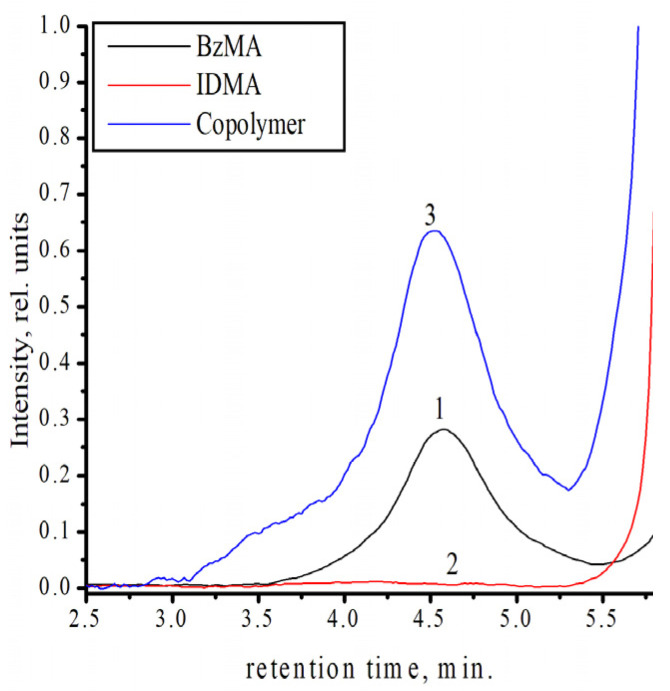
GPC of polymer samples (polymerization time—2 h): (1) polybenzyl methacrylate; (2) polyisodecyl methacrylate; (3) 1:1 copolymer (in moles).

After electron beam treatment of isodecyl methacrylate, benzyl methacrylate, and their equimolar mixture, the samples were kept at a temperature of 50, 60, and 70 °C. The results of the chromatographic analysis showed that no polymerization occurred at a temperature of 50–60 °C for the radiation dose of 20 kGy. Figure 2 shows the chromatograms after 2 h of heat treatment at 70 °C.

It is clear that in the case of isodecyl methacrylate, the amount of polymer produced is insufficient for accurate identification using this method, even with a significant increase in heating duration. In the case of an equimolar mixture, the polymer conversion (3.2%) substantially exceeds the areas of the conversion (1.6%) for benzyl methacrylate. So, we can state that for heat treatment at 70 °C, the rate of mixture polymerization is substantially higher compared to the polymerization of individual monomers. Previously, we determine copolymer homogeneity by the fractional precipitation method [30]. We have shown that the initial copolymer and its isolated fractions were same. Based on that and the result of GPC, we concluded that we indeed produced the copolymer and not a blend of individual polymers. The molecular weight of the copolymer is the same as that of its isolated fractions (M_w_~1,750,000 g/mol), and they have the same degree of polydispersity (M_w_/M_n_~2.89).

For further identification of the polymers and their copolymer, the 1H NMR method was used; the spectra are presented in Figures S1–S6. Tables S1 and S2 show the positions of the proton signals for polyisodecyl methacrylate and polybenzyl methacrylate, respectively. The signals 2.61, 2.81, 5.55–6.10 and 2.61, 2.81, 5.59–6.16 indicate that polymerization is occurring for isodecyl methacrylate and benzyl methacrylate, respectively.

**Figure 3:**
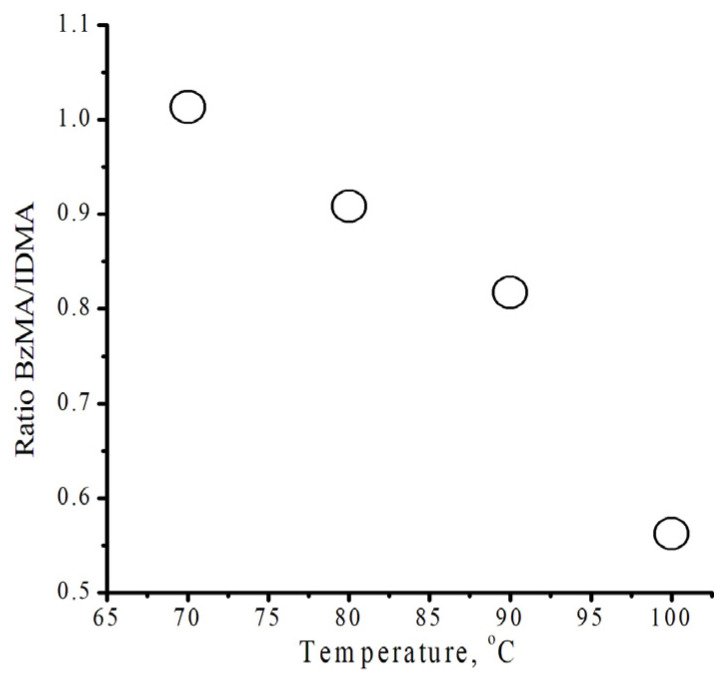
The relationship between the ratio of monomers in a copolymer and polymerization temperature.

We examined the impact of temperature on the copolymer (Figure S5) composition using ^1^H NMR spectroscopy (Figure S6). Based on the obtained ^1^H NMR spectroscopy data, the ratio of the integral intensities of signals from methylene 5.5` groups at 5.2 ppm and 4.26–4.00 ppm (Table S3) was used to calculate the monomer ratio in the copolymer (Formula (1)). Figure 3 shows the temperature dependence of the copolymer unit content at the initial stages of polymerization. At the initial stages of polymerization, when the process is carried out at temperatures below 70 °C, benzyl methacrylate units predominate in the copolymer. As the process temperature increases to 100 °C, isodecyl methacrylate content in the copolymer increases significantly. These data also indicate the presence of sterically stabilized isodecyl methacrylate molecules in the mixture and the influence of this effect on the copolymer composition ratio.

χ_a_ = I_a_ × m_b_/(I_a_ × m_b_ + I_b_ × m_a_) (1)

Formula (1) I_a_, I_b_—integral intensities of monomer signals in the binary copolymer; m_a_, m_b_—number of protons in the corresponding repeating unit for each signal.

We hypothesize that intermolecular interactions in isodecyl methacrylate are impeding the movement of molecules. That may be the cause of the reduced reactivity of individual monomers relative to their combination [33].

### 3.2. No-D NMR Studies of Methacrylic Acid Esters

**Figure 4:**
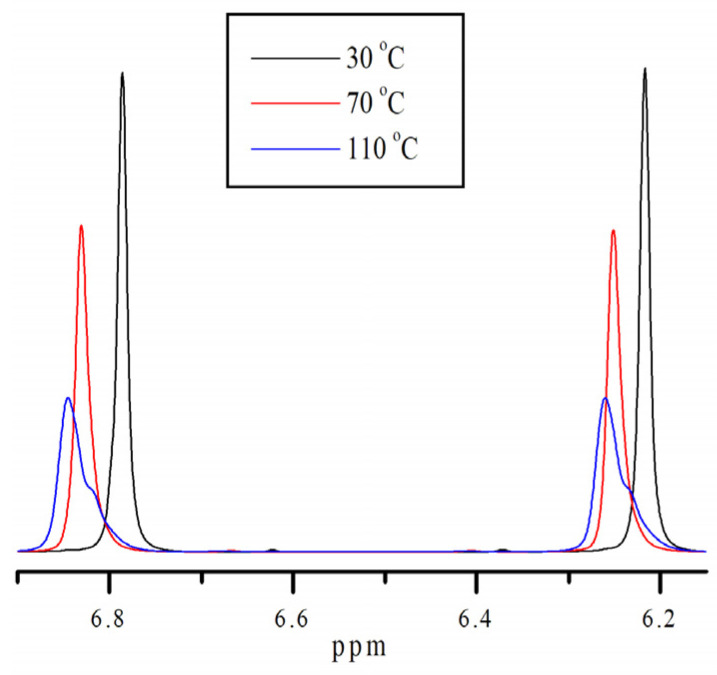
Fragments of No-D ^1^H NMR spectra in situ at 1 in isodecyl methacrylate at different temperatures.

**Figure 5:**
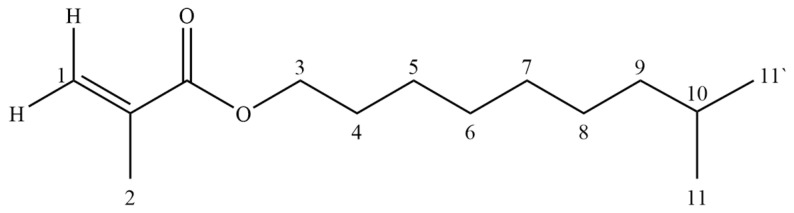
Molecular structures of isodecyl methacrylate.

To confirm the presence of different molecular states in the monomers, additional studies were conducted using no-deuterium proton nuclear magnetic resonance [34,35]. Traditional ^1^H NMR is not applicable in our case, due to the fact that we are interested in intermolecular processes that occur in pure monomers. Figure 4 shows the fragments of the No-D ^1^H NMR in situ spectra (Figure S7) of isodecyl methacrylate (Figure 5), recorded at 30, 70, and 110 °C. The peaks at 6.2–6.9 ppm are attributed to the protons at position 1 of isodecyl methacrylate. The signals at 5.01–4.75 ppm and 2.69 ppm are attributed to the protons at positions 5 and 3, respectively. The remaining signals at 2.54–1.48 ppm are attributed to the methyl and methylene protons of isodecyl groups (Table 1).

According to spectral analysis, temperatures higher than 70 °C result in the identification of a second set of signals corresponding to proton 1 in isodecyl methacrylate. Similarly, at location 1 in benzyl methacrylate, Figures S8 and S9 also introduces a second set of signals from protons. Additionally, the No-D ^1^H NMR in situ signals of benzyl methacrylate were identified (Table 2).

**Figure 6:**
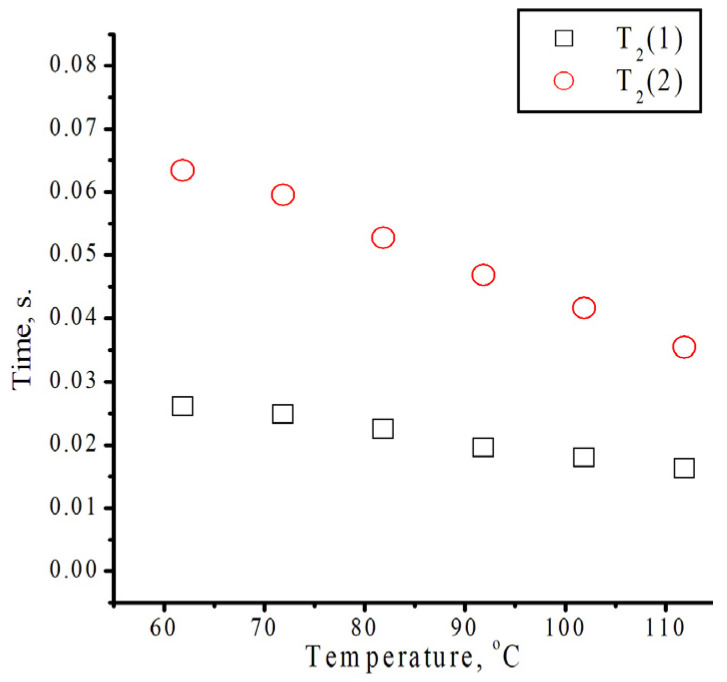
Spin–spin relaxation time for isodecyl methacrylate at different temperatures. 1—calculated by original peak in multipeak; 2—calculated by emerging peak in multipeak.

The spin–spin relaxation time of isodecyl methacrylate was calculated using Formula (2) and displayed as a temperature-dependent curve (Figure 6).

T_2_ = 1/(π × ∆υ_1/2_) (2)

T_2_ is the spin–spin relaxation time (seconds); ∆υ_1/2_ is the linewidth at half the height of the peak (Hz).

We laid out the peaks at 6.2–6.9 ppm according to Gaussian decomposition. The spin–spin relaxation time (T_2_) is the time required for the transverse magnetization to decrease to approximately 1/e of its initial value.

NMR relaxation times reflect both the molecular structure of a substance and its mobility. Our data suggest that changes in relaxation times may be associated with increased molecular mobility in the substance.

**Figure 7:**
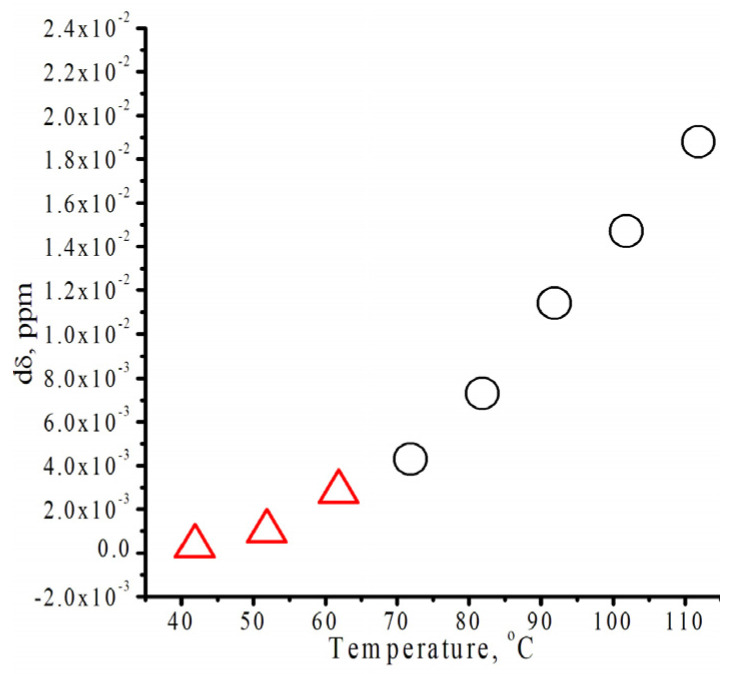
Difference in the positions of proton signals at 1 in the No-D ^1^H NMR spectra in situ of isodecyl methacrylate at different temperatures.

Figure 7 illustrates the effect of temperature on the chemical shift of the proton at 1 in isodecyl methacrylate. The image clearly shows two regions: below and above 70 °C. It is clear that the dependence is linear at temperatures higher than 70 °C.

**Figure 8:**
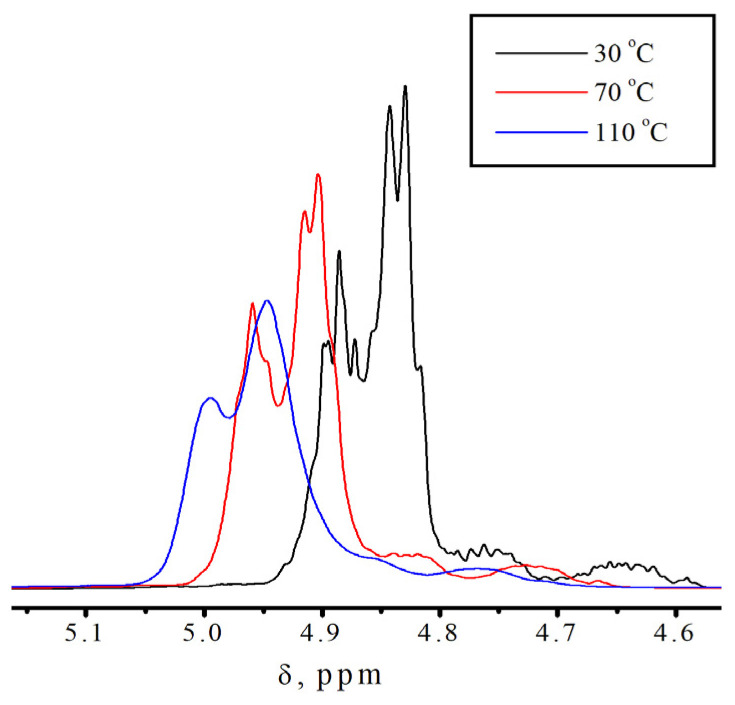
Fragments of No-D ^1^H NMR spectrum in situ of a bulky substituent in isodecyl methacrylate at different temperatures.

The fragments of the ^1^H NMR spectrum in situ, as shown in Figure 8, shows signals from the protons of the bulky substituent in isodecyl methacrylate.

As shown in Figure 8, at temperatures below 70 °C the number of peaks is higher than at elevated temperatures, and these peaks are clearly distinguishable. This behavior of the No-D ^1^H NMR spectrum in situ also indicates the supramolecular arrangement of the monomer.

**Figure 9:**
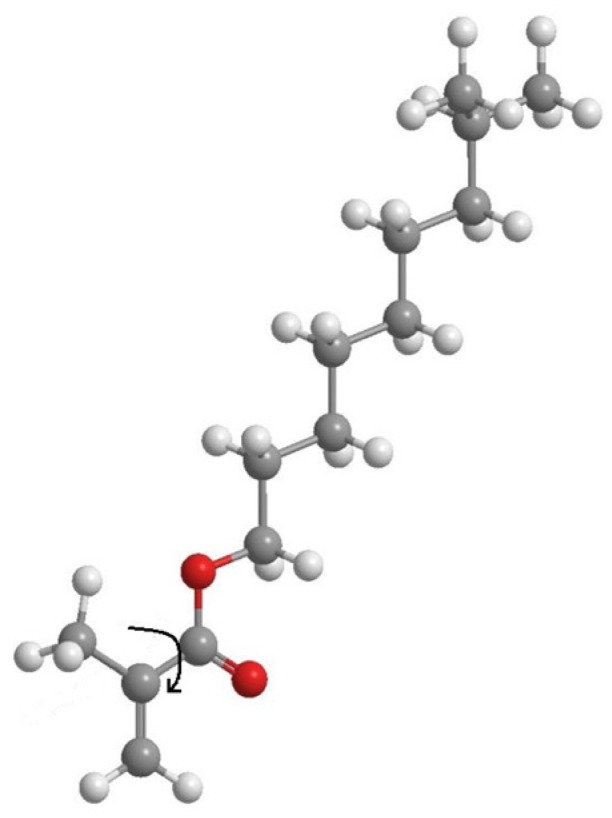
Intramolecular rotation of isodecyl methacrylate.

The supramolecular arrangement of the monomer below 70 °C is indicated by the additional proton peaks for the bulky substituent in isodecyl methacrylate at lower temperatures. This is further demonstrated by the existence of a second set of No-D proton signals at 1 at temperatures higher than 70 °C. A supramolecular structure is associated with the presence of molecular associates as a result of non-covalent intermolecular interactions found only in pure isodecyl methacrylate (Figure S10). Polymerization clearly inhibits supramolecular ordering. In this case, intramolecular rotation is one of the most likely polymerization outcomes (Figure 9). Intramolecular rotation is frequently impeded by potential barriers associated with the presence of intermolecular interactions. The activation energy of this process, determined from the Arrhenius plot, is 7–8 kJ/mol.

Similar experiments with benzyl methacrylate (Figure S11) also confirmed the presence of rotational dynamics upon heating. The onset temperature of the rotational process was 10–15 °C lower than in isodecyl methacrylate. The calculated activation energy for this process was 3.3 kJ/mol.

The activation energies for bulk polymerization via the radical mechanism were derived from literature sources [36]. The activation energies for isodecyl methacrylate and benzyl methacrylate polymerization are 20.8 and 6.83 kJ/mol, respectively. Thus, the activation energy for intramolecular rotation accounts for 40–50% of the activation energy of polymerization.

**Figure 10:**
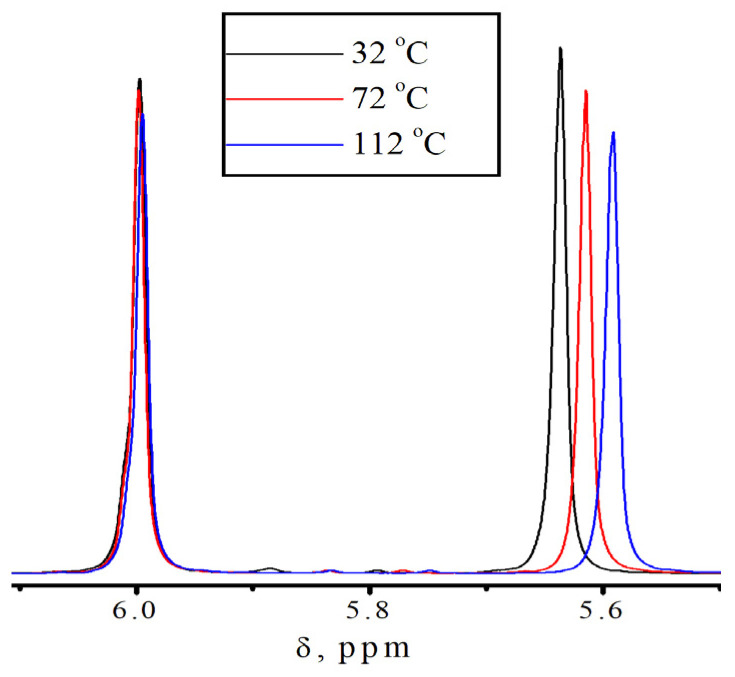
Fragments of No-D ^1^H NMR spectra in situ at 1 in isodecyl methacrylate in DMSO at different temperatures.

**Figure 11:**
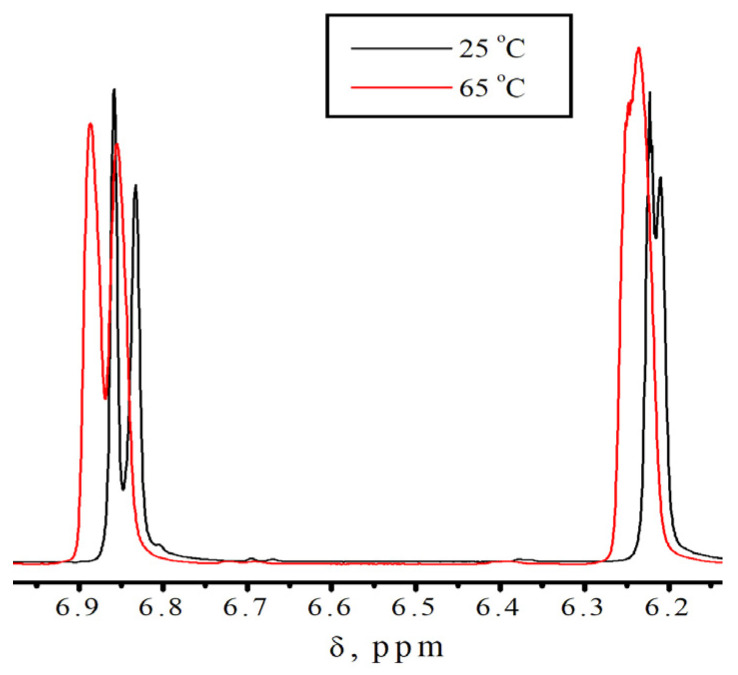
Fragments of No-D ^1^H NMR spectra in situ at 1 in isodecyl methacrylate and benzyl methacrylate in their equimolar solution, recorded at different temperatures.

The fact that this phenomena of the additional proton peaks for 1 (Figure 4) only occurs with pure isodecyl methacrylate is an interesting aspect of it. It is not observed for a solution of isodecyl methacrylate in DMSO (Figure 10 and Figure S11) or for a mixture of isodecyl methacrylate and benzyl methacrylate (Figure 11).

Figure 11 depicts the fragments of the No-D ^1^H NMR spectra in situ of an equimolar mixture of isodecyl methacrylate and benzyl methacrylate. These fragments align with the signals from protons at 1 in isodecyl methacrylate and benzyl methacrylate. The change in the signal shape with an increase in temperature indicates the occurrence of a process that promotes polymerization. Moreover, this process starts to manifest itself at lower temperatures than for the individual monomers.

**Figure 12:**
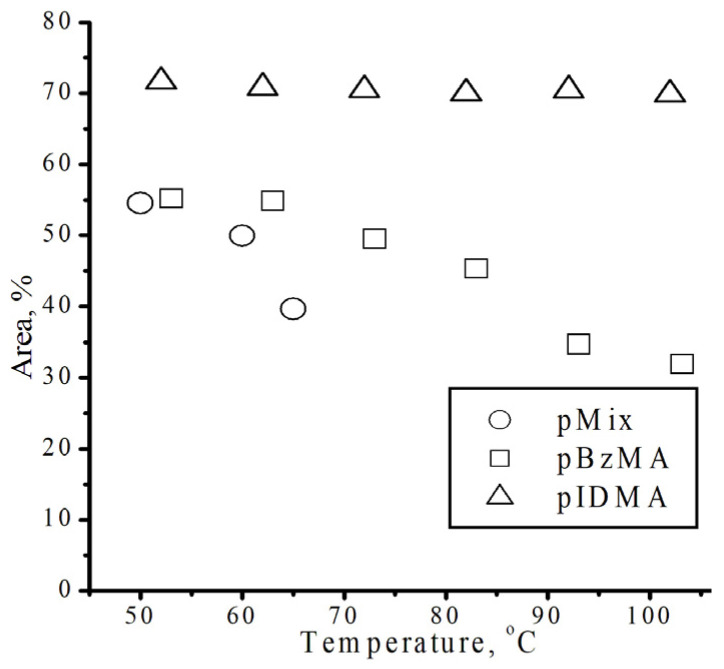
Area of proton peaks No-D ^1^H NMR in situ at 1 isodecyl methacrylate and benzyl methacrylate, and the equimolar solution of isodecyl methacrylate/benzyl methacrylate at different temperatures.

This effect is demonstrated in Figure 12. The Gaussian decomposition of the No-D ^1^H NMR in situ signals at 1 for isodecyl methacrylate, benzyl methacrylate, and a mixture of isodecyl methacrylate and benzyl methacrylate enables us to ascertain the temperature dependence of the reactivity of monomers during polymerization. For isodecyl methacrylate, the peak area and shape exhibit a minor change compared to benzyl methacrylate. At the same time, the peak area for benzyl methacrylate and for the mixture of isodecyl methacrylate and benzyl methacrylate diminishes with increasing NMR spectral temperature due to the appearance of a second set of peaks.

**Figure 13:**
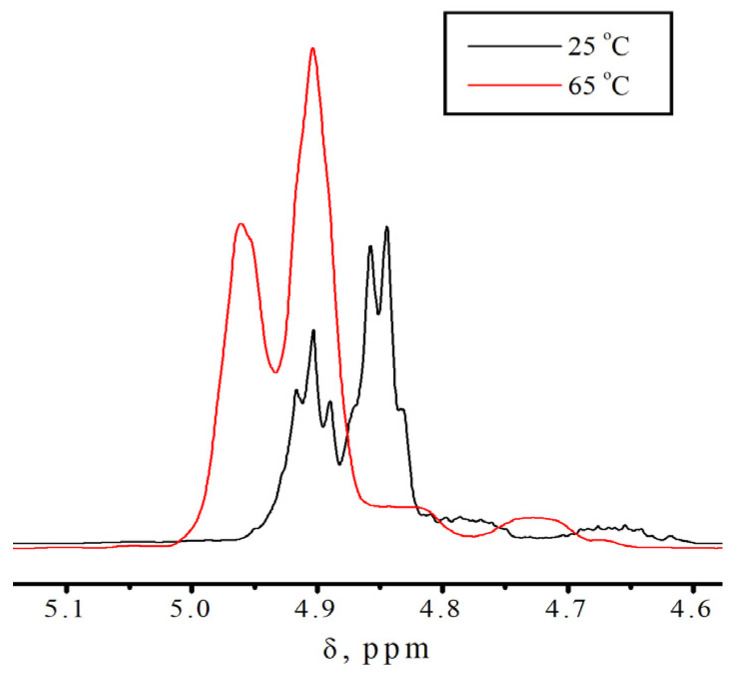
Fragments of No-D ^1^H NMR spectra in situ of a bulky substituent in isodecyl methacrylate/benzyl methacrylate equimolar solution, recorded at different temperatures.

A similar behavior is observed for the proton signals of the bulky isodecyl methacrylate substituent in an equimolar solution of isodecyl methacrylate with benzyl methacrylate (Figure 13). Potential barriers exist, likely due to the supramolecular arrangement, just like in a pure monomer. The main difference in the behavior of an equimolar solution of isodecyl methacrylate with benzyl methacrylate from isodecyl methacrylate alone (Figure S10) is that in the case of a monomer mixture, the removal of potential barriers occurs at lower temperatures.

### 3.3. Studies of Physical Parameters of Monomers and Their Mixtures

**Figure 14:**
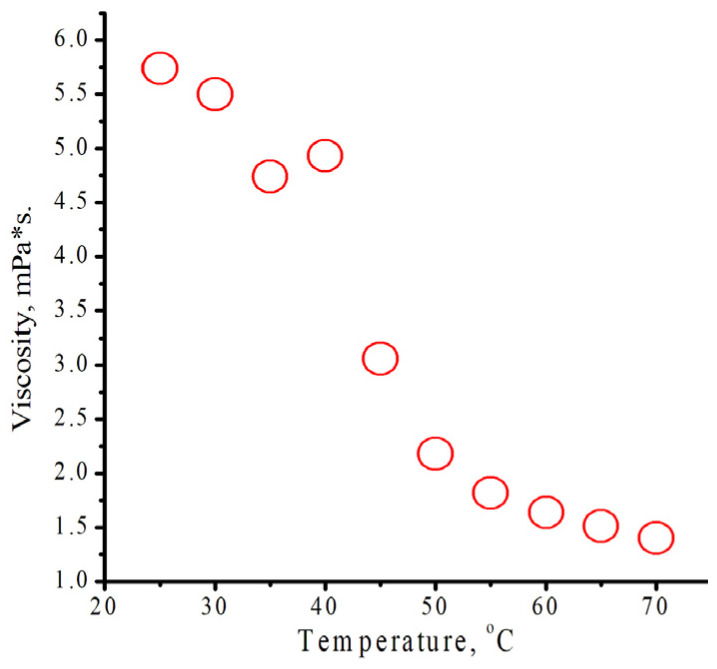
Dependence of the viscosity of isodecyl methacrylate on temperature.

To determine the nature of this phenomenon, we analyzed a number of physical characteristics of monomers. The presence of “frozen” molecules in the monomer indirectly validates the temperature dependence of the viscosity of individual monomers and their mixtures. Initially, at the same temperature, the viscosity of isodecyl methacrylate (Figure 14) is higher than that of benzyl methacrylate (Figure S12), but it drops sharply upon heating and becomes lower than that of benzyl methacrylate. That behavior might take place due to the presence of intermolecular interactions of isodecyl methacrylates (Figure 4). Although the viscosity of the mixture (Figure S13) decreases more gradually than that of individual monomers, its viscosity is less than that of individual monomers. The inflection point on Figure 14 could indicate a transition between different molecular states in isodecyl methacrylate.

Using Formula (3), the activation energy for the viscous flow of benzyl methacrylate is calculated at 13.30 kJ/mol. Other calculated data are presented in Table 1. Values for isodecyl methacrylate were determined using the data from the straight sections of the graphs. Depending on the graph section, activation energy data may fluctuate within the error thresholds. The average values of parameters are shown in Table 3. As shown in Table 1, the activation energy for the viscous flow of equimolar mixture of monomers is lower than the activation energy of pure monomers. This data also indicates the presence of “frozen” molecules in the monomer.

η = A × exp(Ea/(R × T))(3)

**Figure 15:**
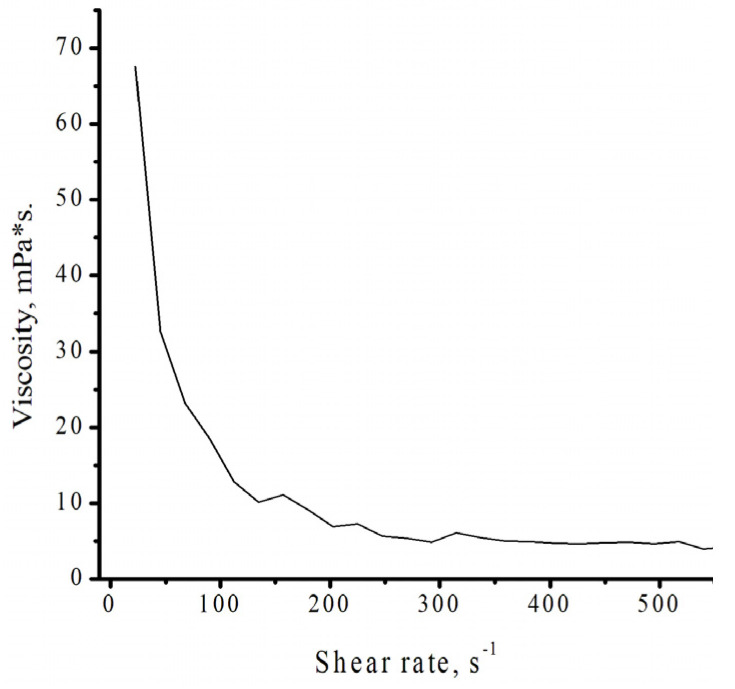
Dependence of the viscosity of isodecyl methacrylate on the shear rate at a temperature of 40 °C.

As shown in Figure 15, isodecyl methacrylate is classified as a pseudoplastic fluid. Pseudoplastic fluids are time-independent non-Newtonian fluids whose viscosity decreases with increasing shear rate [37]. This group is the most numerous and perhaps the most important class of non-Newtonian fluids. Paint systems, printing inks, and dispersed systems in general provide familiar examples. In such systems, the viscosity–shear relationship is crucial for process requirements, and a high degree of pseudoplasticity is generally undesirable, as it implies inadequate flow at low shear rates. Precise knowledge of the viscosity–shear relationship is also important both as a practical foundation for rheological characterization and for the fundamental comprehension of non-Newtonian behavior [38].

**Figure 16:**
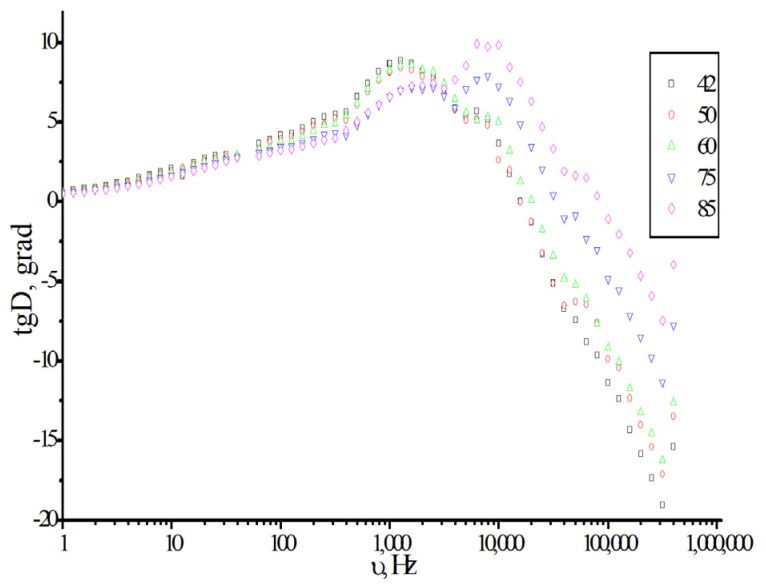
The dielectric loss spectrum of isodecyl methacrylate at different temperatures (^o^C).

To confirm the presence of different molecular states in the monomers, additional studies were conducted using dielectric spectroscopy. Figure 16 shows the frequency dependence of dielectric loss curves recorded at different temperatures.

The dielectric loss spectrum in isodecyl methacrylate exhibits two peaks at 2.5 and 10 kHz at temperatures exceeding 60 °C. This indicates the presence of at least two molecular states within the monomer [39]. Heating causes a partial transition from the initial state, presumably consisting of monomer associates, to a more disordered state, in which a higher mobility of the monomers and their fragments is observed. The new maximum dielectric loss is at ~10 kHz.

**Figure 17:**
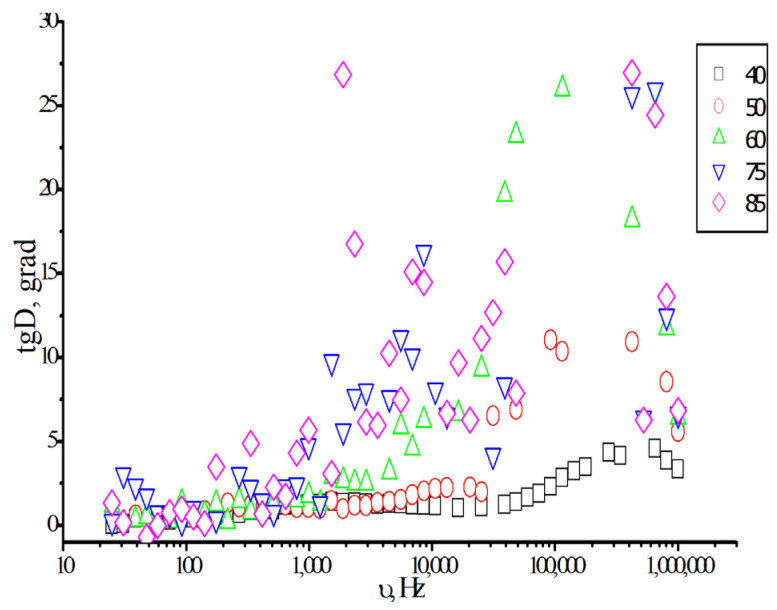
Dielectric loss spectrum of benzyl methacrylate at different temperatures (^o^C).

A single molecular state is observed in benzyl methacrylate, according to the dielectric loss spectrum (Figure 17). As the temperature rises, the dielectric loss in benzyl methacrylate increases, indicating an increase in molecular mobility. At temperatures higher than 75 °C, dielectric loss in benzyl methacrylate is not reliable anymore. Nevertheless, there is no transition between different molecular states.

**Figure 18:**
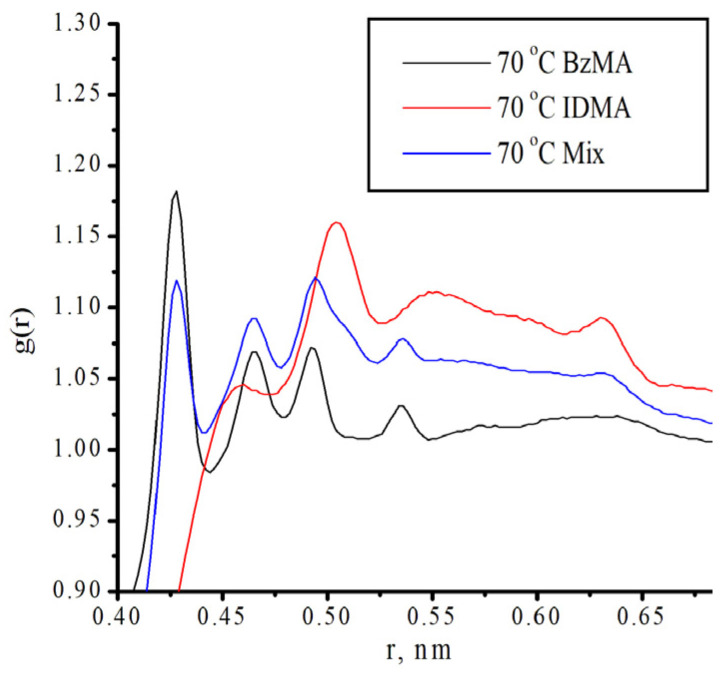
Radial distribution of molecules at a temperature of 70 °C.

**Figure 19:**
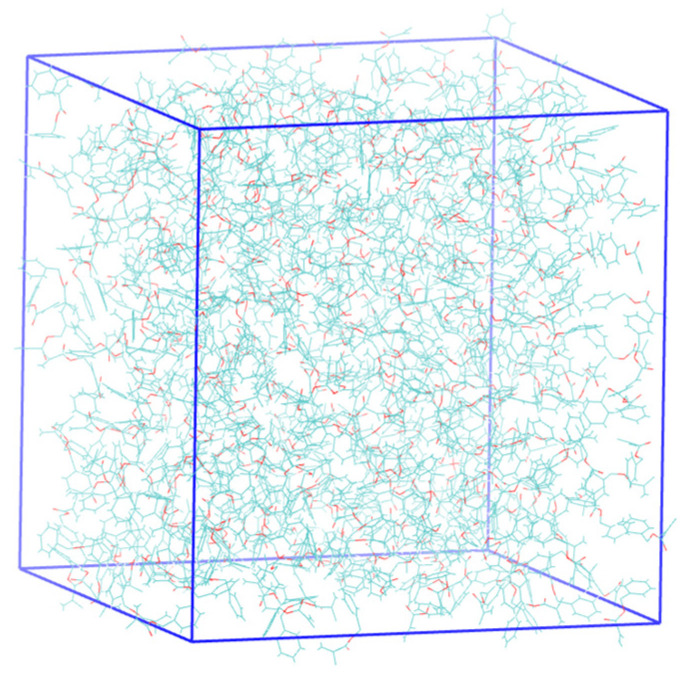
Three-dimensional model of the arrangement of benzyl methacrylate molecules.

Figure 18 and Figures S14–S16 show the dependence of the radial distribution of molecules on the distance to the molecular center. As seen in Figure 18, for the monomer mixture, the number of neighboring molecules is less than for pure monomers. At the same time, the space around benzyl methacrylate is more densely packed than that of isodecyl methacrylate. Despite this, benzyl methacrylate is more reactive than isodecyl methacrylate. This behavior can be explained by the fact that, although the benzyl methacrylate molecules are located close to each other, the reactive centers are not shielded (Figure S15 and Figure 19).

Figures S17 and S18 present the information on the Lennard–Jones energy and Coulomb interaction energy of a monomer mixture. The Lennard–Jones potential is the potential energy of two atoms or free molecules interacting with each other, depending on the distance between them. Coulomb interaction energy is the potential energy between two electrically charged particles, defined by Coulomb’s Law, representing the work needed to separate them. Molecular dynamics simulations can be used to determine various physical characteristics of materials relying on the interaction potentials. According to these data, the Lennard–Jones potential increases with temperature, indicating an increase in the distance between molecules. The change in the Coulomb interaction energy is negligible.

## 4. Conclusions

We demonstrated that the rate of polymerization in monomer mixtures is substantially higher than in pure monomers. It has been established that isodecyl methacrylate polymerization does not occur at temperatures below 70 °C. We confirmed the hypothesis that polymerization does not occur due to the substantial degree of association of the liquid monomer, hindering structural rearrangements. It is shown that this phenomenon is observed solely for pure isodecyl methacrylate, but not for its solutions or equimolar mixtures with benzyl methacrylate.

## Figures and Tables

**Table 1:** Chemical shift of No-D ^1^H in the NMR spectrum in situ 30 °C of isodecyl methacrylate.

Atom number	1	2	3	4–10	11, 11`
Chemical shift, ppm	6.78; 6.21	2.69	5.04–4.60	2.55–1.83	1.68

**Table 2:** Chemical shift of No-D ^1^H NMR in situ 30 °C in benzyl methacrylate.

Atom number	1	2	3	4, 4`, 5, 5`, 6
Chemical shift, ppm	6.60–5.70	2.30	5.55	7.77–7.52

**Table 3:** Parameters obtained from Equation (1).

Sample	Pre-Exponential Factor	Ea, kJ/mol
Isodecyl methacrylate	8.7 ± 0.7	16.4 ± 0.2
Benzyl methacrylate	9.7 ± 0.3	13.3 ± 0.1
Equimolar mixture of monomers	9.8 ± 0.2	12.8 ± 0.06

## Data Availability

The authors declare that the data supporting the findings of this study are available within the paper. Should any raw data files be needed in another format they are available from the corresponding author upon reasonable request.

## Supplementary Materials

The following supporting information can be downloaded at: https://www.mdpi.com/article/10.3390/polym18030368/s1: Figure S1. Molecular structures of isodecyl methacrylate and benzyl methacrylate; Figure S2. The ^1^H NMR spectra in CDCl_3_ of poly (isodecyl methacrylate) obtained at a temperature of 70 ^o^C; Figure S3. Molecular structures of isodecyl methacrylate and benzyl methacrylate; Figure S4. The ^1^H NMR in CDCl_3_ spectra of poly (benzyl methacrylate) obtained at a temperature of 70 ^o^C; Figure S5. Molecular structures of isodecyl methacrylate and benzyl methacrylate; Figure S6. The ^1^H NMR in CDCl_3_. spectra of copolymer of isodecyl methacrylate and benzyl methacrylate obtained at a temperature of 70 ^o^C; Figure S7. The No-D ^1^H NMR spectra of isodecyl methacrylate in situ at 30^o^C; Figure S8. The No-D ^1^H NMR spectra of benzyl methacrylate in situ at 30^o^C; Figure S9. Fragments of No-D ^1^H NMR spectra at 1 in benzyl methacrylate in situ at different temperatures; Figure S10. Molecular structures of benzyl methacrylate; Figure S11. Fragments of No-D ^1^H NMR spectra of bulky substituent in isodecyl methacrylate in DMSO in situ at different temperatures; Figure S12. Dependence of the viscosity of benzyl methacrylate on temperature; Figure S13. Dependence of the viscosity of equimolar mixture of monomers on temperature; Figure S14. Radial distribution of isodecyl methacrylate molecules; Figure S15. Radial distribution of benzyl methacrylate molecules; Figure S16. Radial distribution of molecules in mixture of monomers; Figure S17. Lennard–Jones energy of a mixture of monomers at different temperatures; Figure S18. Energy of Coulomb interaction in a mixture of monomers at different temperatures; Table S1. Chemical shift of ^1^H in the NMR spectrum of poly (isodecyl methacrylate); Table S2. Chemical shift of ^1^H in the NMR spectrum of poly (benzyl methacrylate); Table S3. Chemical shift of ^1^H in the NMR spectrum of copolymer;

## Abbreviations

The following abbreviations are used in this manuscript:

THF	Tetrahydrofuran
INP SB RAS	Budker Institute of Nuclear Physics Siberian Branch of the Russian Academy of Sciences
NMR	Nuclear Magnetic Resonance
GPC	Gel permeation chromatography
