# Supplementary material for: Characterization of Electron Beam-Induced Polymerization of Isodecyl Methacrylate, Benzyl Methacrylate, and Their Equimolar Mixture Based on Monomer Properties
Source: Polymers (Basel). 2026 Jan 29;18(3):368. doi: 10.3390/polym18030368 (PMC12899341; doi:10.3390/polym18030368)
Supplement: Supplementary file 1 [file polymers-18-00368-s001.zip › polymers-4027698-supplementary.pdf]

Supplemental materials

Ilia Antonov [1,*], Mikhail Mikhailenko [1], Tatyana Shakhtshneider [1] , Artem Ulihin [1], Maxim Zelikman [1] , Alexandr Bryazgin [2], Ilia Eltsov [3]

[1] Institute of Solid State Chemistry and Mechanochemistry SB RAS, Novosibirsk, 630090 Russian Federation; secretary@solid.nsc.ru
[2] Budker Institute of Nuclear Physics SB RAS, Novosibirsk, 630090 Russian Federation; inp@inp.nsk.su
[3] Novosibirsk State University, Novosibirsk, 630090 Russian Federation; interstudy@lab.nsu.ru
* Correspondence: iliya.antonov92@yandex.ru

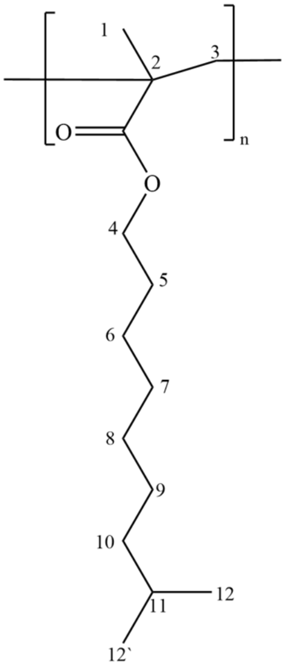

Figure S1. Molecular structures of isodecyl methacrylate and benzyl methacrylate.

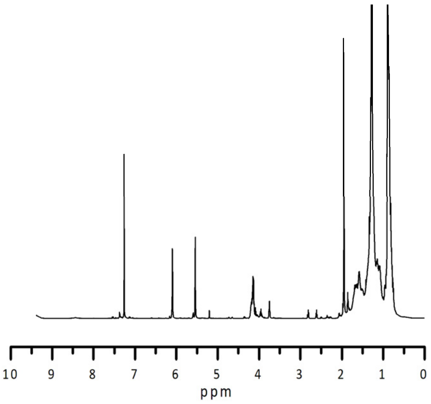

Figure S2. The ^{1}H NMR spectra in $CDCl_3$ of poly(isodecyl methacrylate) obtained at a temperature of 70 ^{o}C.

Table S1. Chemical shift of ¹H in the NMR spectrum of poly(isodecyl methacrylate)

Atom number	2(at chains end)	3(at chains end)	3	4	1, 5-11	12, 12′
Chemical shift, ppm	2.61; 2.81	5.55-6.10	1.98	4.10-4.23	1.04-1.46	0.84

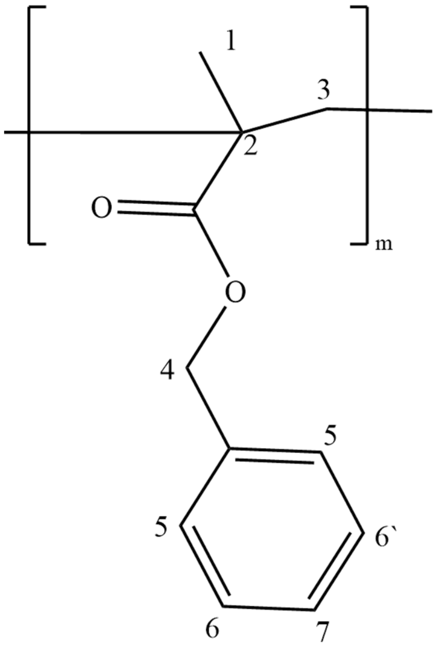

Figure S3. Molecular structures of isodecyl methacrylate and benzyl methacrylate.

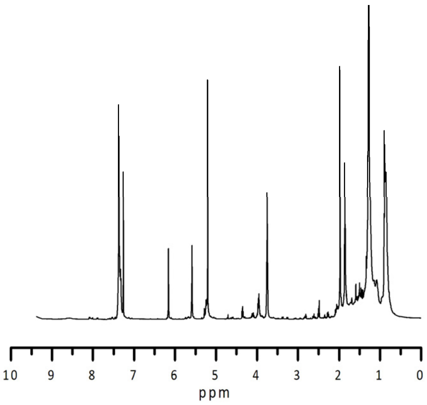

Figure S4. The ¹H NMR in $CDCl_3$ spectra of poly(benzyl methacrylate) obtained at a temperature of 70 °C.

Table S2. Chemical shift of ¹H in the NMR spectrum of poly(benzyl methacrylate)

Atom number	1	2(at chains end)	3(at chains end)	3	4	5, 5′, 6, 6′, 7
Chemical shift, ppm	0.88-1.24	2.61; 2.81	5.59-6.16	1.84-1.97	5.20	7.22-7.44

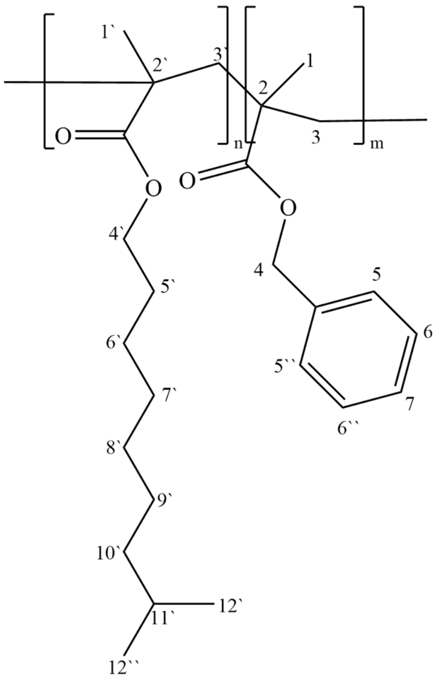

Figure S5. Molecular structures of isodecyl methacrylate and benzyl methacrylate.

Table S3. Chemical shift of ^{1}H in the NMR spectrum of copolymer.

Atom number	2, 2`(at chains end)	3, 3`(at chains end)	3, 3′	4	4′	6, 6′′, 7, 7′′, 8	1, 1′, 3, 3′, 5′-12′, 12′′
Chemical shift, ppm	2.61; 2.81	5.55-6.16	1.98	5.2	4.22-4.05	7.44-7.22	2.09-0.8

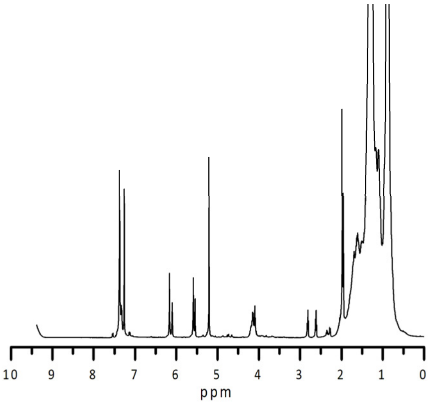

Figure S6. The ^{1}H NMR in $CDCl_3$. spectra of copolymer of isodecyl methacrylate and benzyl methacrylate obtained at a temperature of 70 ^oC.

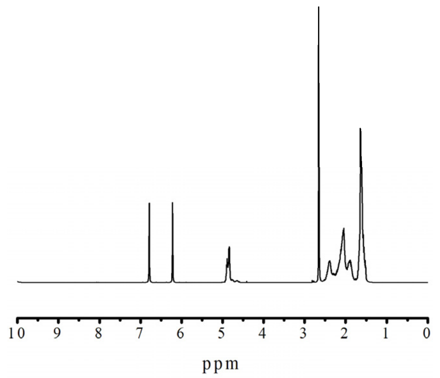

Figure S7. The No-D ^1H NMR spectra of isodecyl methacrylate in situ at 30°C.

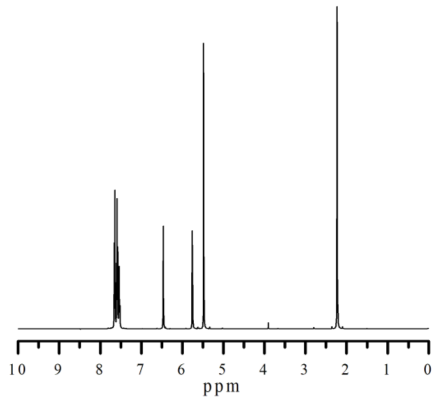

Figure S8. The No-D ^1H NMR spectra of benzyl methacrylate in situ at 30°C.

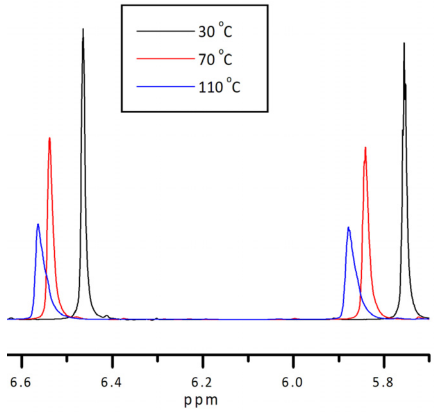

Figure S9. Fragments of No-D ^1H NMR spectra at 1 in benzyl methacrylate in situ at different temperatures.

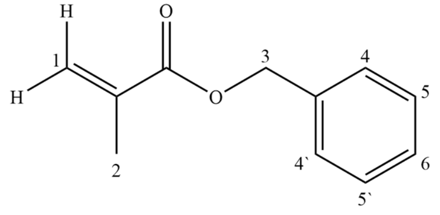

Figure S10. Molecular structures of benzyl methacrylate.

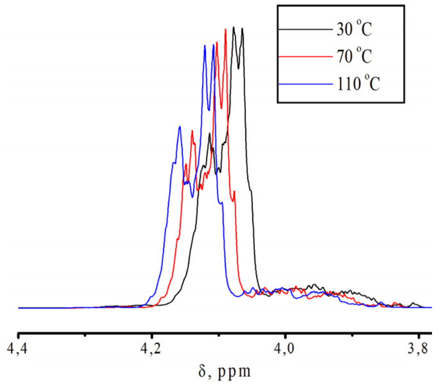

Figure S11. Fragments of No-D ^{1}H NMR spectra of bulky substituent in isodecyl methacrylate in DMSO in situ at different temperatures.

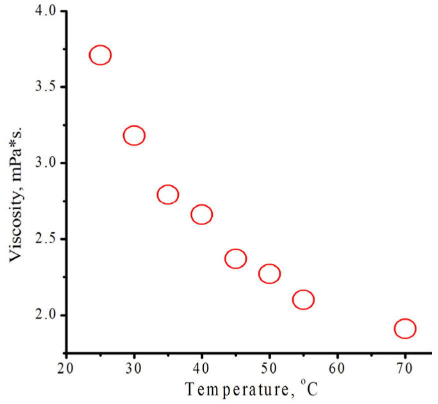

Figure S12. Dependence of the viscosity of benzyl methacrylate on temperature.

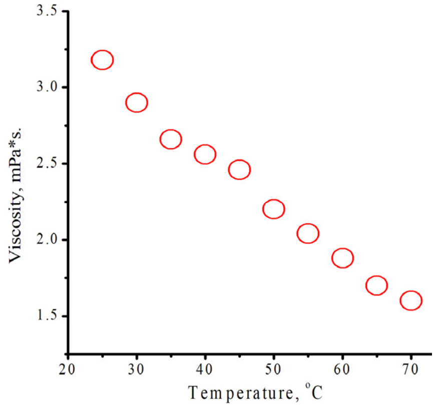

Figure S13. Dependence of the viscosity of equimolar mixture of monomers on temperature.

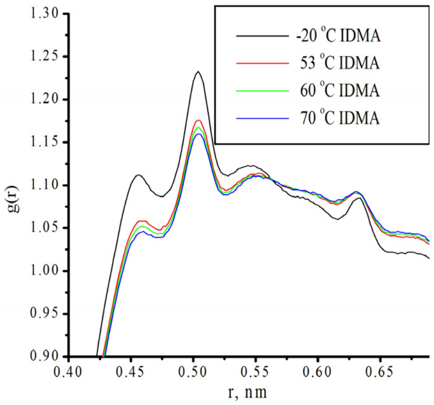

Figure S14. Radial distribution of isodecyl methacrylate molecules.

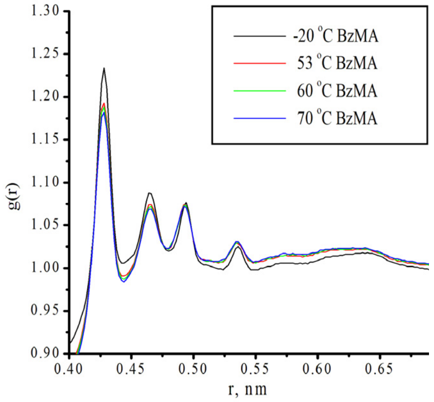

Figure S15. Radial distribution of benzyl methacrylate molecules.

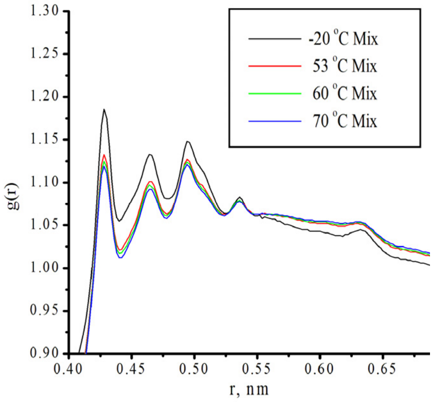

Figure S16. Radial distribution of molecules in mixture of monomers.

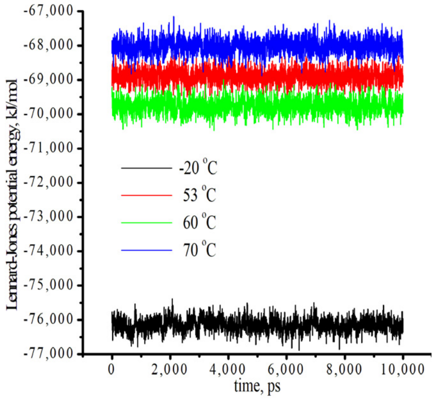

Figure S17. Lennard–Jones energy of a mixture of monomers at different temperatures.

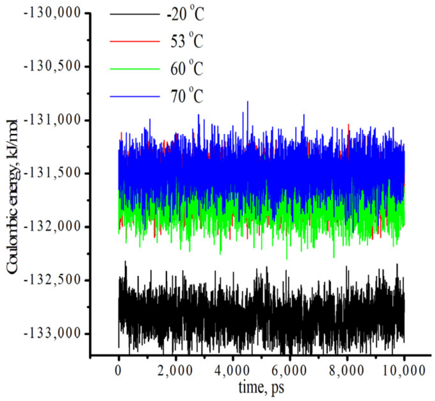

Figure S18. Energy of Coulomb interaction in a mixture of monomers at different temperatures.
